# Resolving single membrane fusion events on planar pore-spanning membranes

**DOI:** 10.1038/srep12006

**Published:** 2015-07-13

**Authors:** Lando L. G. Schwenen, Raphael Hubrich, Dragomir Milovanovic, Burkhard Geil, Jian Yang, Alexander Kros, Reinhard Jahn, Claudia Steinem

**Affiliations:** 1 Institute for Organic and Biomolecular Chemistry, University of Göttingen, Tammannstr. 2, 37077 Göttingen, Germany; 2 Max-Planck-Institute for Biophysical Chemistry, Am Fassberg 11, 37077 Göttingen, Germany; 3 Institute for Physical Chemistry, University of Göttingen, Tammannstr. 6, 37077 Göttingen, Germany; 4 Leiden Institute of Chemistry - Supramolecular and Biomaterials Chemistry, Leiden University, Einsteinweg 55, 2333 CC Leiden, The Netherlands

## Abstract

Even though a number of different *in vitro* fusion assays have been developed to analyze protein mediated fusion, they still only partially capture the essential features of the *in vivo* situation. Here we established an *in vitro* fusion assay that mimics the fluidity and planar geometry of the cellular plasma membrane to be able to monitor fusion of single protein-containing vesicles. As a proof of concept, planar pore-spanning membranes harboring SNARE-proteins were generated on highly ordered functionalized 1.2 μm-sized pore arrays in Si_3_N_4_. Full mobility of the membrane components was demonstrated by fluorescence correlation spectroscopy. Fusion was analyzed by two color confocal laser scanning fluorescence microscopy in a time resolved manner allowing to readily distinguish between vesicle docking, intermediate states such as hemifusion and full fusion. The importance of the membrane geometry on the fusion process was highlighted by comparing SNARE-mediated fusion with that of a minimal SNARE fusion mimetic.

In most physiological fusion processes, a strongly to moderately curved membrane (e.g. synaptic vesicle, virion) fuses with a planar membrane (e.g. (neuronal) plasma membrane). Despite these geometric considerations of the fusing membranes in nature, most *in vitro* results on fusion have been obtained from bulk vesicle assays, where two curved membranes fuse with each other resulting in an increase in vesicle size, i.e. the membrane’s curvature changes during the fusion process^1^,^2^,^3^,^4^,^5^. In order to start already with a planar membrane geometry, either giant unilamellar vesicles (GUVs)^6^,^7^, freestanding black lipid membranes^8^,^9^ or membranes attached to a solid support have been used^10^,^11^,^12^,^13^,^14^. While GUVs allow only for bulk measurements, black lipid membranes lack mechanical and long-term stability and are thus not well suited. Supported membranes are unique as they are very robust and enable one to detect individual fusion events using total internal reflection fluorescence (TIRF) microscopy^10^,^14^,^15^,^16^. Observing membrane fusion at the single-particle level provides several advantages over bulk vesicle assays. Single-vesicle based experimental designs overcome problems such as vesicle aggregation, and bursting, and provide access to the intermediate states during membrane fusion^17^,^18^,^19^. These intermediate states are not accessible in bulk fusion assays, where only an average kinetics is monitored and individual and rare events are hidden^20^. However, disadvantages of supported lipid membranes are that, owing to the close contact of the target planar membrane with the support, they lack a second aqueous compartment, do not provide enough space for the incoming lipid material, and show a reduced lateral mobility of the membrane components, in particular of proteins. The mobility issue can be partly improved by decoupling the membrane from the support. For example, Wagner *et al.*^21^ introduced a polyethyleneglycol spacer between membrane and substrate, which resulted in an increased mobility of the reconstituted proteins.

As there are still a number of drawbacks associated with current state-of-the-art membrane architectures used for investigating fusion processes, there is a demand for improved and alternative systems overcoming these disadvantages. Thus, we aimed at a planar membrane architecture that is long-term stable and provides laterally mobile membrane components similar to the situation *in vivo*. Moreover, this membrane system should allow for the detection of single vesicle fusion events, should be accessible from both sides and should provide enough space for the incoming lipid material during the fusion process. One membrane system that might suffice these requirements are pore-spanning membranes^22^,^23^,^24^,^25^. Pore-spanning membranes have been shown to be mechanically robust and long-term stable^26^, and the lipids in the pore-spanning membrane regions are laterally mobile^27^. As they are deposited on an open pore array, both aqueous compartments can be addressed individually^28^. In a previous study, we have shown that single-vesicle fusion can be observed on pore-spanning membranes providing sufficient space for the additionally incoming lipid material^29^. However, in this study, pore-spanning membranes, termed nano-black lipid membranes, prepared from painting lipids dissolved in an organic solvent were used, which do not allow for protein reconstitutions.

Hence, it was the aim of this study to develop a pore-spanning membrane system with reconstituted fusion proteins to be able to develop a new *in vitro* fusion assay devoid of organic solvent. We established a method to reconstitute neuronal SNARE proteins into pore-spanning membranes, which enabled us to monitor individual fusion events between a pore-spanning membrane and SNARE-containing large unilamellar vesicles.

SNARE proteins are the well-known key players in neuronal membrane fusion and an excellent test case for the development and evaluation of the envisioned membrane fusion system. In synaptic transmission three SNARE proteins, namely syntaxin 1, SNAP25 and synaptobrevin 2 mediate the fundamental steps of membrane fusion^30^. Syntaxin 1 and SNAP25 are localized in the mainly planar presynaptic membrane, with which synaptobrevin 2 containing synaptic vesicles can fuse. To mimic this *in vivo* situation, we established a reconstituted membrane system as depicted in Fig. 1A. SNARE complexes composed of syntaxin 1A, SNAP25a and a soluble fragment of synaptobrevin 2 are embedded in a planar pore-spanning membrane by spreading SNARE-containing giant unilamellar vesicles (GUVs) on a gold-coated functionalized regular pore array in silicon nitride with pore diameters of 1.2 μm (Fig. 1B), while the fusing vesicles contain synaptobrevin 2. The success of pore-spanning membrane formation can be readily observed by fluorescence microscopy with a characteristic fluorescence pattern (Fig. 1C). The fluorescence pattern arises from the fact that the fluorescence of the membrane is quenched on the gold covered pore rims owing to the close contact of the membrane to the support. No quenching occurs in the freestanding parts of the membrane resulting in the pore pattern in the fluorescence images, which allows to observe each individual pore-spanning membrane^31^.

## Results

### SNARE reconstitution into pore-spanning membranes and lateral mobility of the components

**Figure 2:**
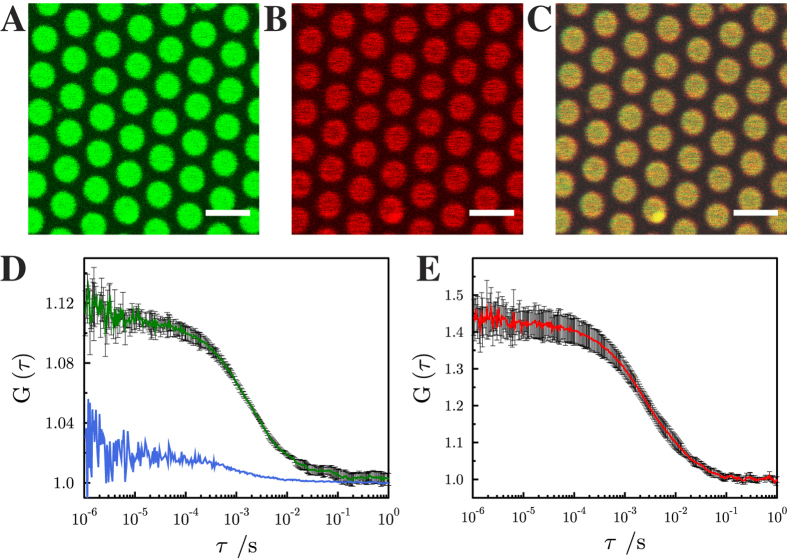
Fluorescence micrographs of pore-spanning membranes composed of DOPC/POPE/POPS/cholesterol (5:2:1:2) on a 6-mercapto-1-hexanol functionalized gold covered porous silicon nitride surface. The images show fluorescence signals of (**A**) Atto488 DPPE, (0.01 mol%), (**B**) Atto647N-syntaxin 1 transmembrane domain (0.0055 mol%) and (**C**) an overlay of (**A**) and (**B**). Scale bars: 3 μm. Autocorrelation curves of the performed FCS measurements with excitation wavelengths of 488 nm (Atto488 DPPE, green, **D**) and 633 nm (Atto647N-syntaxin 1-TMD, red, **E**). The blue FCS curve in D was obtained on a gold covered pore rim showing that the fluorescence of Atto488 DPPE is significantly quenched and hence, the diffusion constant cannot be determined. Fitting eq. (4) to the autocorrelation curves provide diffusion coefficients of 7.7 ± 0.4 μm^2^/s (SD) for Atto488 DPPE and 3.4 ± 0.2 μm^2^/s (SD) for Atto647N-syntaxin 1-TMD. For Atto488-DPPE *L* was 200 nm, and for Atto647N-syntaxin 1A *L* was 250 nm. For each diffusion constant, at least 20 pore-spanning membranes from three independent reconstitutions were analyzed.

Spreading of a GUV to form pore-spanning membranes is achieved by a proper functionalization of the porous silicon nitride substrate. The highly ordered pore array made of Si3N4 with pore diameters of 1.2 μm is gold coated at the top surface followed by functionalization with 6-mercapto-1-hexanol^32^. To examine whether the SNARE proteins reconstituted into GUVs according to the protocol described in the Methods Section, are successfully transferred into pore-spanning membranes, two different fluorescently labeled syntaxin derivatives were used. A synthetic transmembrane domain (TMD) labeled with Atto647N (Atto647N-syntaxin 1-TMD) as previously used for GUV experiments^33^, and a cysteine mutant of syntaxin 1A labeled with Alexa488 (Alexa488-syntaxin 1A). Both were reconstituted into GUVs composed of 1,2-dioleoyl-*sn*-glycero-3-phosphocholine (DOPC)/1-palmitoyl-2-oleoyl-*sn*-glycero-3-phosphoethanolamine (POPE)/1-palmitoyl-2-oleoyl-*sn*-glycero-3-phospho-L-serine (POPS)/cholesterol (5:2:1:2). After spreading of the GUVs, the fluorescence of the Atto647N-syntaxin 1-TMD (Fig. 2B) as well as that of Alexa488-syntaxin 1A (Supplementary Fig. 1B) was readily observed in the pore-spanning membranes indicating successful reconstitution of transmembrane peptides in pore-spanning bilayers. The fluorescence intensity of Atto647N-syntaxin 1-TMD was fully homogeneous throughout the free-standing parts of the pore-spanning membranes. However, the fluorescence of Alexa488-syntaxin 1A appeared to be slightly inhomogeneous, which might be explained by the tendency of syntaxin 1A to cluster, driven by homotypic protein-protein interactions^34^,^35^.

Prerequisite for the formation of a fusion complex during fusion of a vesicle with the planar bilayer is the unhindered lateral movement of the syntaxin transmembrane helices in the plane of the pore-spanning membrane. Previously, we have shown that our membrane architecture ensures lipid mobility in both leaflets^36^. However this does not necessarily imply that transmembrane proteins are fully mobile^21^,^37^. In case of solid supported bilayers, which also provide a planar membrane geometry, one lipid leaflet is attached to a support even if a polymer cushion or lipid tethers are added to the system^20^, which can considerably influence the lateral mobility of the proteins. To investigate the diffusion behavior of the incorporated syntaxin 1A transmembrane helix in the pore-spanning membrane, fluorescence correlation spectroscopy (FCS) was performed using Atto647N-syntaxin 1-TMD as well as Alexa488-syntaxin 1A together with two differently labeled lipids to investigate a conceivable influence of the fluorescent dye on the diffusion coefficient.

From the autocorrelation curves, a diffusion constant of 7.7 ± 0.4 μm^2^/s (SD) for the lipid Atto488 DPPE (Fig. 2D) and 7.4 ± 0.3 μm^2^/s (SD) for DPPE-KK114 was determined (Supplementary Fig. 1D) demonstrating dye independent lipid diffusion constants. For the transmembrane peptide Atto647N-syntaxin 1-TMD a diffusion constant of 3.4 ± 0.2 μm^2^/s (SD) was found, (Fig. 2E), while the diffusion constant for Alexa488-syntaxin 1A was determined to be 2.3 ± 0.2 μm^2^/s (SD) (Supplementary Fig. 1E). The determined diffusion constants of the lipids and syntaxin domains are in agreement with those determined in freestanding membranes of GUVs^38^,^39^ and black lipid membranes (BLMs)^40^. The smaller diffusion constants of syntaxin 1-TMD and syntaxin 1A compared to the lipids complies with the expectation taking the slightly larger molecular area and the transmembrane helix spanning both lipid leaflets of the membrane into account. In conclusion, our results clearly demonstrate that the membrane components are laterally mobile in the freestanding parts of the pore-spanning membranes, a prerequisite for the formation of fusion active SNARE complexes.

Measuring the lipid and protein diffusion in membrane areas on the solid support, however, was not feasible due to quenching of the fluorescence by the underlying gold layer (Fig. 2D, blue curve). The question if the incorporated membrane components are mobile beyond the rims of the individual pores was answered by fluorescence loss in photobleaching (FLIP) experiments. Here, the fluorescence was bleached for a long time period in a region of interest on a pore-spanning membrane patch leading to a loss of fluorescence in the entire membrane patch (Supplementary Fig. 2 and 3). This result demonstrates that lipids and proteins can diffuse from one pore-spanning membrane to the other. However, a reduced diffusion of the membrane components is expected in the membrane areas attached to the pore rims compared to the freestanding parts. For example, Przybylo *et al.*^39^ determined a more than two times slower diffusion constant for lipids in solid supported membranes compared to diffusion constants found in vesicles.

### Detection of SNARE-mediated single vesicle fusion events with pore-spanning membranes

For the detection of single vesicle fusion events with pore-spanning membranes, suspended membranes composed of DOPC/POPE/POPS/cholesterol (5:2:1:2) containing reconstituted preassembled 1:1:1 syntaxin 1A-SNAP25a-synaptobrevin 2 (residues 49-96) complexes (1,000:1), termed the ΔN-acceptor complex^5^, were prepared by GUV spreading. Large unilamellar vesicles were composed of DOPC/POPE/POPS/cholesterol (5:2:1:2) with reconstituted full length synaptobrevin 2 (1,000:1) exhibiting a mean diameter of 325 ± 20 nm as determined by dynamic light scattering. The functionality of the reconstituted SNARE proteins, i.e. the fusogenic activity and specificity of large unilamellar vesicles (LUVs) with reconstituted synaptobrevin 2 and GUVs containing the ΔN-acceptor complex, was proven by a standard bulk vesicle fusion assay (Supplementary Fig. 4).

**Figure 3:**
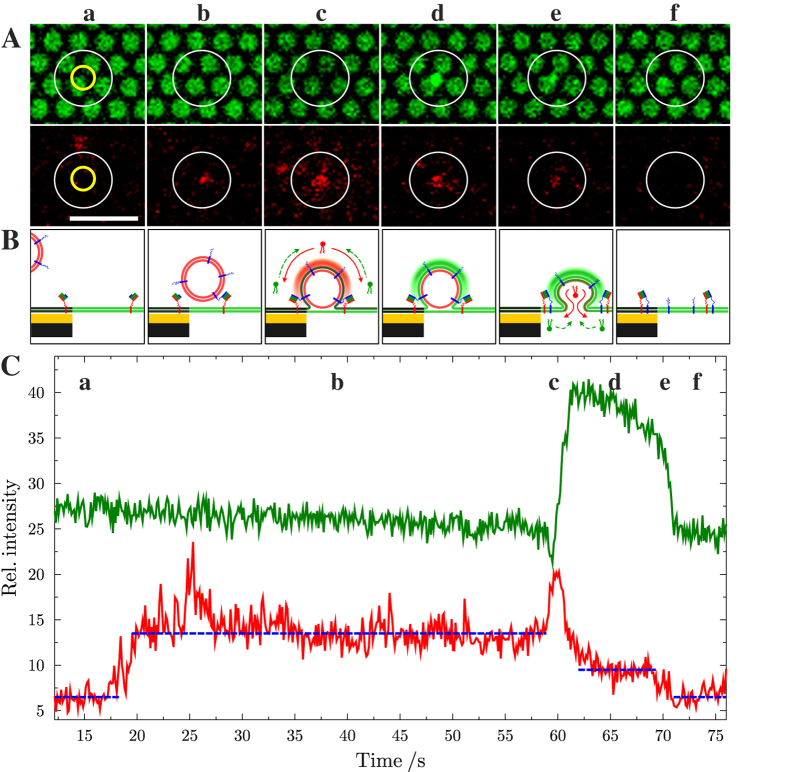
(**A**) Time lapse series of fluorescence micrographs showing a single fusion event of a large unilamellar vesicle containing full length synaptobrevin 2 with a pore-spanning membrane with reconstituted ΔN-acceptor complex. (top) Oregon Green DHPE fluorescence of the pore-spanning membranes; (bottom) Texas Red DHPE fluorescence of the vesicle. The yellow circles in (**a**) shows the region of interest (ROI) that was used for reading out the time-resolved changes in the relative fluorescence intensities depicted in (**C**); the white circles in the images highlight the region, where the vesicle docks and fuses and only serves as a guide to the eye. Scale bar: 5 μm. (**B**) Schematic drawing of the postulated fusion states at the time of image recording. Membranes are colored according to their incorporated fluorescent dye. (**C**) Time resolved changes in the relative fluorescence intensity of Oregon Green DHPE (green) and Texas Red DHPE (red) during the shown fusion event. Dashed blue lines serve as a guide to the eye highlighting the distinct levels of intensity. Letters (**a**–**f**) correspond to the fluorescence images in (**A**). For further details see text and Supplementary Fig. 7.

**Figure 4:**
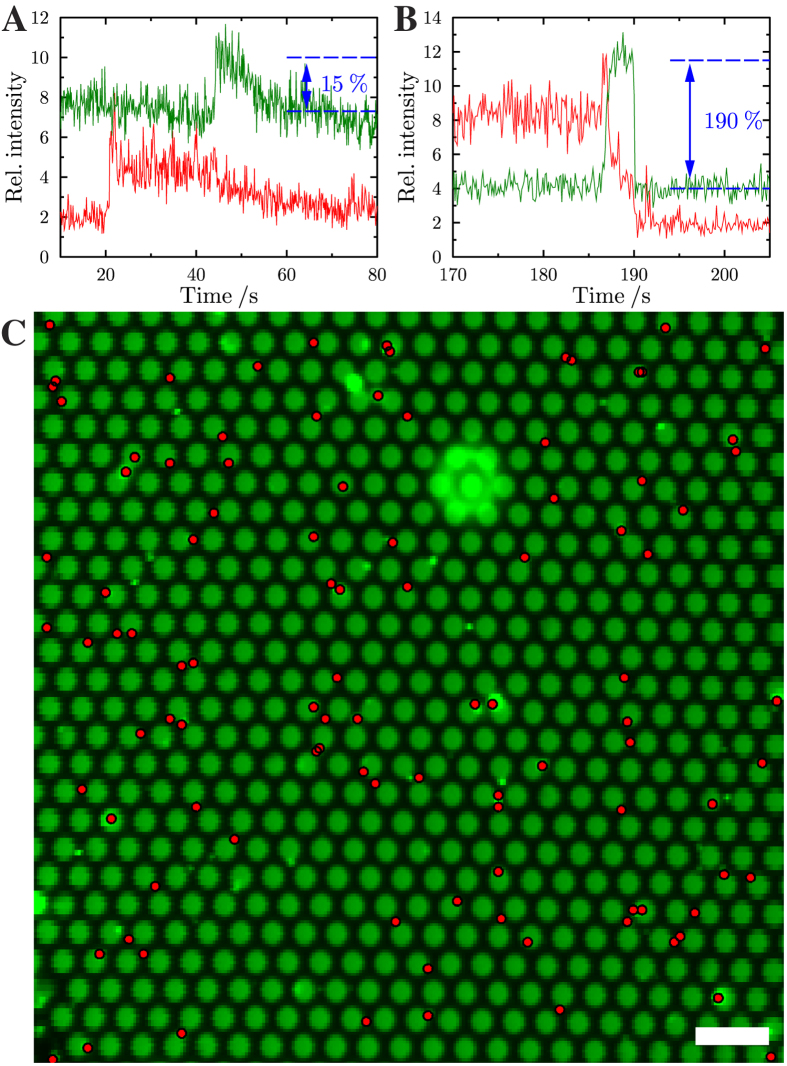
Time resolved changes in fluorescence intensity of Oregon Green DHPE (green) and Texas Red DHPE (red) during (**A**) a fusion event occurring in the center of the pore-spanning membrane and (**B**) occurring with a pore-spanning membrane attached to the pore rim. (**C**) Averaged Oregon Green fluorescence image. The red dots mark the positions of the centers of mass of the docked vesicles. Scale bar: 4 μm.

Pore-spanning membrane patches of various sizes ranging from 10–150 μm in diameter were obtained by GUV spreading as visualized by the Oregon Green DHPE fluorescence (Fig. 3A,a). For the detection of single vesicle fusion events, membrane patches with a size larger than 40 × 40 μm^2^ were selected on the porous surface. After addition of full length synaptobrevin 2 containing LUVs doped with Texas Red DHPE, time series for both lipid dyes were recorded simultaneously (Supplementary Movie 1). A characteristic time series of movie frames of a single vesicle fusion event that basically captures all observables in these experiments is depicted in Fig. 3. The time dependent fluorescence intensity changes of Oregon Green DHPE and Texas Red DHPE upon docking and fusion of a single vesicle to the planar membrane was monitored by defining a region of interest (ROI, yellow colored circle in Fig. 3A,a) around the center of mass of the docked vesicle and integrating the fluorescence intensity as a function of time. The obtained time traces of the two fluorophores including all characteristic features of a single vesicle fusion event are depicted in Fig. 3C. Prior to vesicle docking a low Texas Red DHPE (TR) and a high Oregon Green DHPE (OG) fluorescence is observed (Fig. 3C,a) When a single vesicle approaches the surface and docks, a sudden increase in TR fluorescence occurs, while the OG fluorescence remains unaffected (Fig. 3C,b). Upon onset of fusion (Fig. 3C,c) the TR intensity shows a transient increase, while concomitantly a small transient decrease in the OG fluorescence intensity is observed, which is a result of the Förster resonance energy transfer (FRET) between the TR and OG dye as the lipids of the outer leaflet of the vesicle (Texas Red DHPE) start mixing with the lipids of the outer leaflet of the pore-spanning membrane (Oregon Green DHPE) (Fig. 3C,c). After this first transient changes of TR and OG fluorescence, the TR fluorescence intensity drops to and then remains at an intensity that is well above baseline level but lower than that of the originally docked vesicle. Simultaneously, an increase in OG fluorescence intensity is observed. These fluorescence time traces can be explained with the formation of an intermediate hemifused state (Fig. 3C,d). In the hemifused state, the outer leaflets merge, while the inner leaflet of the TR-doped vesicle remains unaffected. Thus, after a first merger of the outer leaflets, the TR fluorescence intensity drops to an intermediate intensity level, as Texas Red DHPE lipids located in the outer leaflet of the vesicle diffuse into the pore-spanning membrane. Simultaneously, Oregon Green DHPE lipids diffuse into the outer leaflet of the vesicle and move away from the porous surface into the third dimension resulting in an increase in OG fluorescence intensity. The magnitude of the observed OG fluorescence increase depends on the position of the fusing vesicle. Fig. 4 shows two single fusion events at two different positions of the pore-spanning membrane. If the vesicle fuses in the center of the pore-spanning membrane (Fig. 4A) only a small increase in fluorescence intensity is observed as a result of the three dimensional vesicle structure atop the pore-spanning membrane at the hemifusion state. From geometric considerations taking the average membrane area of the outer membrane leaflet of the vesicle (~0.3 μm^2^) and the area of both leaflets of the pore-spanning membrane in the ROI (~1.5 μm^2^) into account, an OG intensity increase of about 20% is expected, which agrees well with the observed increase in OG fluorescence (Fig. 4A). However, if the vesicle fuses at the pore rim, where the OG fluorescence of the planar membrane is quenched owing to the close contact with the underlying gold surface^31^, the OG fluorescence gets significantly increased. This is a result of the Oregon Green DHPE lipids diffusing into the outer leaflet of the vesicle with a dimension of several hundred nanometers. As the OG lipids move significantly away from the gold covered surface, fluorescence quenching, which occurs at distances within about 15 nm from the gold surface, becomes almost zero^31^. Instead de-quenching and surface-enhanced fluorescence is observed, which is found at tens of nanometers beyond the range of quenching^41^ resulting in an increase in OG fluorescence significantly larger than the calculated 20% (Fig. 4B).

As shown in Fig. 3C,d the OG fluorescence does not remain constant during the intermediate state but slowly decreases. This is not always but frequently observed and is discussed in terms of a shrinkage of the fusing vesicle (Fig. 3B,e). A similar behavior was observed by Yoon *et al.*^17^. They monitored lipid mixing of distinct vesicle pairs and found a stepwise process of lipid mixing throughout the course of the fusion process, which they assigned to multiple intermediate states. Such intermediate states, i.e. hemifusion and flickering of fusion pores allows lipid material to get slowly or stepwise released into the pore-spanning membrane resulting in a shrinkage of the fusing vesicle. Shrinkage of synaptic vesicles in living cells has also been observed in the process of repeated kiss-and run fusion^42^,^43^. Upon fusion of the inner membrane leaflets (Fig. 3), both fluorescent dye intensities drop to the baseline level (Fig. 3f) indicating full fusion of both membranes.

The FRET in the OG fluorescence time traces (transient decrease in OG fluorescence) was only observed in about 4% of the cases. It was mainly observed if large vesicles fuse, as in most cases the time resolution was not sufficient to resolve the opposing effects of the FRET resulting in OG quenching and diffusion of the OG molecules away from the gold surface, resulting in de-quenching and fluorescence enhancement. Based on the observed fluorescence time traces of OG and TR, we defined criteria to distinguish between only docked vesicles, vesicles that remain in the hemifused state and those that fully fuse (Supplementary Fig. 7).

The specificity of the single vesicle fusion events was verified by several control experiments. First, GUVs were preincubated with the soluble SNARE binding motif of synaptobrevin 2 (residues 1-96) and then spread to form pore-spanning membranes. These pore-spanning membranes did not produce any efficient docking with full length synaptobrevin 2 containing LUVs (Supplementary Movie 2). Second, in the absence of SNAP25a, no fusion was observed.

As the planar pore-spanning membranes consist of solid supported as well as freestanding membrane areas, we further asked the question, if there is a preference for the vesicles to dock. A different behavior is conceivable as the diffusion of the membrane components is expected to be reduced on the pore rims. To analyze this, a grid was computed from the OG fluorescence of the pore-spanning membranes showing the freestanding membrane areas (Fig. 4C). The center of mass positions of each individual docked vesicle obtained by the particle tracking algorithm (see Methods) were then overlaid on this grid. Evaluating 2030 docked vesicles revealed that 27% of the docked vesicles were positioned on the freestanding membrane part that makes up 42% of the entire membrane, which is 64% of the theoretically expected value assuming non-preferential docking. This preference for the vesicles to dock to the solid supported membrane might be a result of increased van der Waals attraction at the gold covered pore rims, which is further enhanced by the conformal contact of the vesicle at the slightly curved border between rim and pore.

### Detecting intermediate states of SNARE-mediated fusion

**Figure 5:**
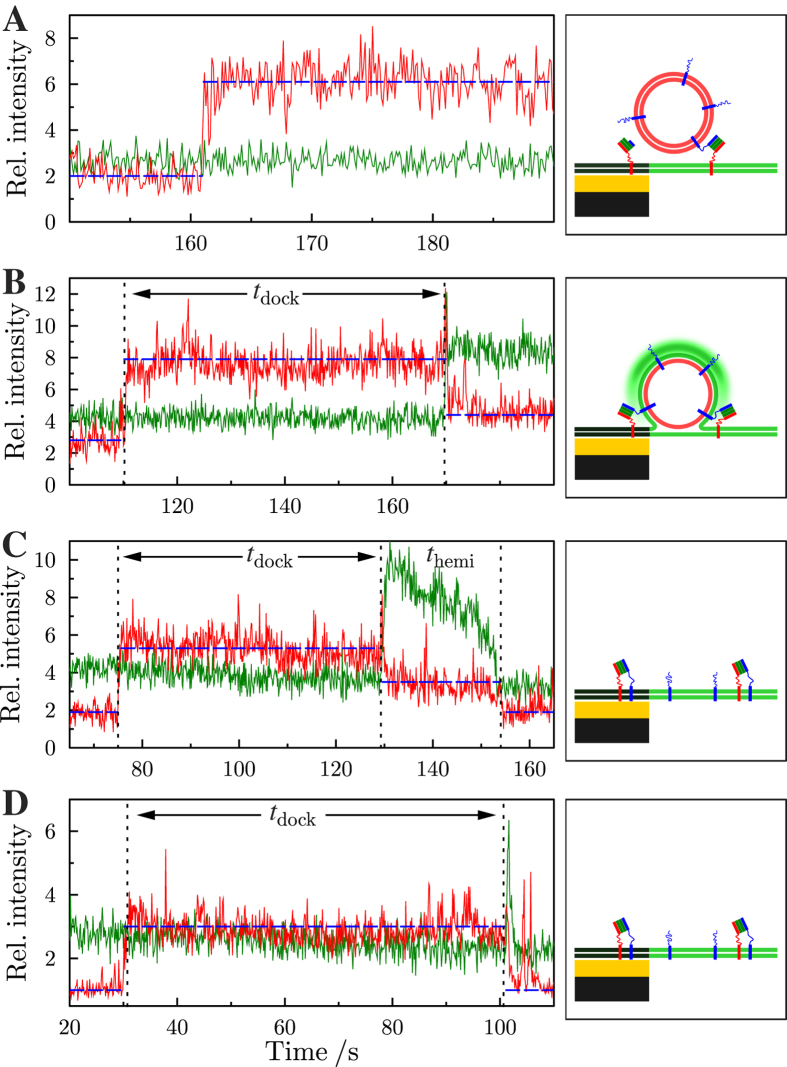
left: Time resolved intensity courses of Oregon Green DHPE (green) and Texas Red DHPE (red). (**A**) Docking of a vesicle, (**B**) hemifusion event, (**C,D**) full fusion events (**C**) with and (**D**) without resolved hemifusion state. The dotted vertical lines illustrate the docking time (*t*_dock_) and hemifusion time (*t*_hemi_). The horizontal dashed blue lines serve as guide to the eye highlighting the distinct levels of intensity. right: Schematics of the scenarios envisioned at the end of the time series. For further details see also Supplementary Fig. 7.

To obtain statistically meaningful data, all vesicles docked onto the pore-spanning membranes were automatically detected by a tracking algorithm, counted and the time traces of the fluorescence intensities were read out from the ROI with 3 pixels around the center of mass of each docked vesicle. As demonstrated in Fig. 3, three different states of the vesicle can be distinguished (Fig. 5A–D, Supplementary Fig. 7). If the TR fluorescence intensity increases in one step to a constant higher value while there is no change in OG fluorescence, the vesicle has only docked (Fig. 5A, docking). The vesicle then either remains docked onto the membrane until the end of the time series or detaches within the observation period indicated by a drop in TR fluorescence intensity in one step to baseline level. Characteristic for the onset of a fusion event following docking of the vesicle is in all cases a simultaneous decrease in TR and an increase in OG fluorescence intensity even if the FRET peak of the OG fluorescence is not observable (Supplementary Fig. 7).

Generally, we distinguish three cases: i) If the TR fluorescence intensity decreases to an intermediate level and does not reach the baseline level, while the OG fluorescence increases and remains high, the vesicle arrests in an intermediate, presumably hemifused state (Fig. 5B, hemifusion). The vesicle can proceed from the intermediate state to the fully fused state indicated by a simultaneous decrease in TR and OG intensity to baseline level. ii) This can occur after the vesicle has been stalled in an intermediate state for a while (Fig. 5C, full fusion) or iii) the vesicle proceeds within 1-2 frames (≈100–200 ms) to a fully fused state (Fig. 5D, full fusion).

We define the time period between vesicle docking and the onset of fusion as docking time (*t*_dock_). The time period between fusion of the outer membrane leaflet, i.e. hemifusion and the inner leaflet, i.e. full fusion, is defined as hemifusion time (*t*_hemi_) (Supplementary Fig. 7). Vesicles that were already docked at the time when the analysis was started were not counted in docking time analysis.

**Figure 6:**
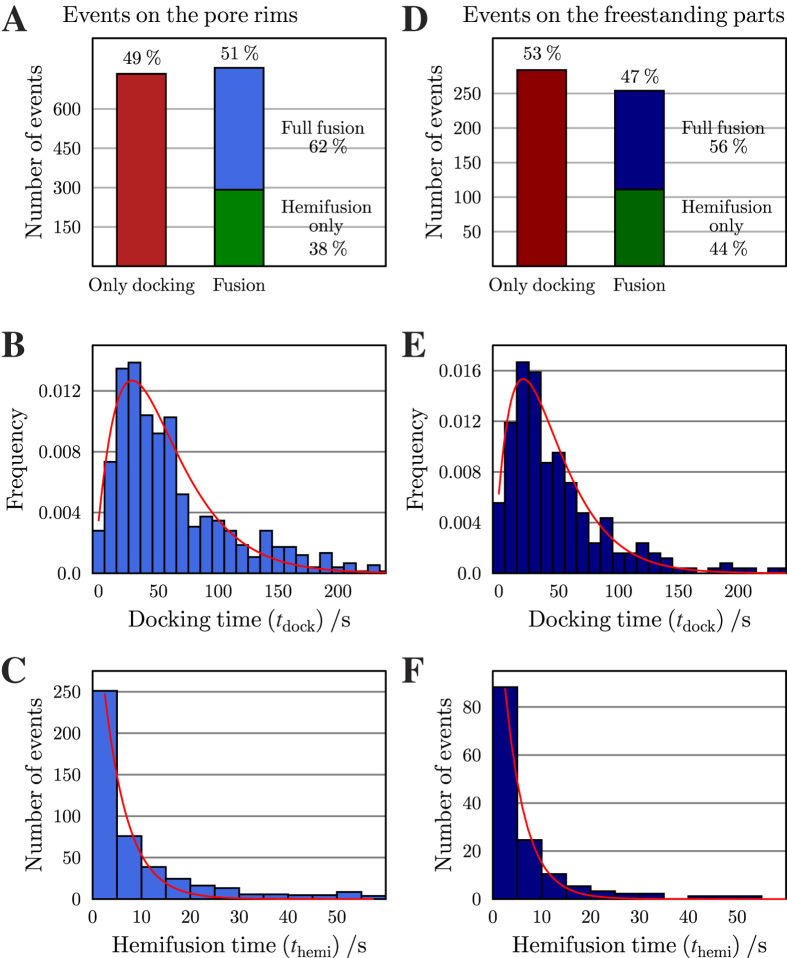
Statistical analysis of the fusion events on the pore rim attached membranes (**A–C**) and on the freestanding parts (**D–F**). **(A**,**D**) Number of vesicles that only dock, dock and fuse but stay in the hemifused state and vesicles that fully fuse with the membrane. (**B**,**E**) Histogram of the docking time *t*_dock_. The red lines are results of fitting eq. (1) to the data. (**C**,**F**) Histogram of the hemifusion time *t*_hemi_. The red line is the result of fitting eq. (2) to the data.

For the statistical analysis, 2030 single vesicle events from 48 time series on individual membrane patches and 10 different reconstitutions were analyzed. We distinguished those events that were observed on the pore rims and those that were found on the freestanding parts of the pore-spanning membranes. 1492 docked vesicles (100%) were found on the pore rims and analyzed, from which 51% progressed to fusion. 62% of the fusing vesicles proceeded to full fusion, while the remaining 38% stayed in an intermediate hemifused state (Fig. 6A). Almost the same results were obtained for those events observed on the freestanding membrane parts. From 538 docked vesicles (100%) on the freestanding parts of the pore-spanning membranes, 47% progressed to fusion, while 53% remained in the docked state. 56% of the fusing vesicles proceeded to full fusion, while 44% remained in the hemifused state (Fig. 6D). From the vesicles that only docked, ⅓ detached during the observation window of 300 s, while ⅔ remained docked during the entire time period. These results indicate that even though there might be a difference in the diffusion behavior of the membrane components within the freestanding pore-spanning membranes and those on the pore rims, this does not considerably influence the ratios of the different observed fusion states.

The negligible impact of the substrate on the fusion efficiency is also reflected in the results found by Tamm and coworkers given that the membrane is sufficiently decoupled from the substrate^14^,^21^. They reported that 43% of all docked vesicles fuse with a planar supported membrane that was detached from the substrate by a polyethyleneglycole spacer to ensure lateral mobility of the SNAREs. This as well as our detected efficiency is much larger than what has been observed by Fix *et al.*^10^. They found an efficiency of only 0.4% in the absence of Ca^2+^, and 15% in the presence of Ca^2+^, even though Ca^2+^ is not required in the minimal fusion system as long as synaptotagmin is not present.

### Kinetics of single fusion events

To shed some light on the fusion kinetics we followed a general model proposed by Floyd *et al.*^16^. In this model, the rate-limiting step from the docked state towards the intermediate state is not a single, one-step transition but a series of *N* transitions between the initial and the intermediate hemifused state with a single rate constant *k*_1_ for each transition leading to eq. (1):
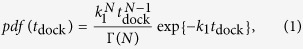
with 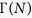 being the gamma function. Using this expression to fit the dwell time distributions of docked vesicles results in a rate constant of *k*_1_ = 0.033 ± 0.003 s^−1^ (SD) with *N* = 1.9 ± 0.2 for those fusion events occurring on the pore rims (Fig. 6B). Fitting eq. (1) to the data plotted in Fig. 6E results in a rate constant of *k*_1_ = 0.035 ± 0.005 s^−1^ (SD) and *N* = 1.7 ± 0.2. For those cases, where the docked vesicles progress to full fusion, a second rate constant *k*_2_ was determined using eq. (2):
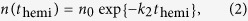
Fitting eq. (2) to the histogram (Fig. 6C) results in a rate constant of *k*_2_ = 0.21 ± 0.02 s^−1^ while *k*_2_ was determined to be 0.24 ± 0.01 s^−1^ for the events detected on membranes on the freestanding part (Fig. 6F). These results indicate that the time constants *k*_1_ and *k*_2_ are almost independent of the position, where the vesicle docks and fuses on the pore-spanning membrane.

### Comparison of SNARE-mediated fusion with SNARE fusion mimetics

**Figure 7:**
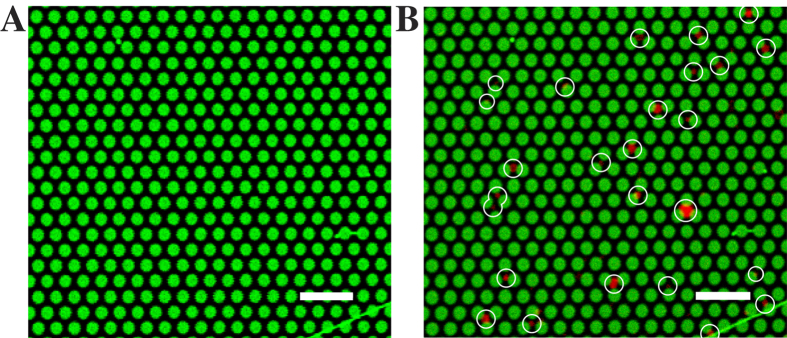
Fluorescence micrographs of a pore-spanning membrane patch containing cholesterol-PEG_12_-(EIAALEK)_4_ (**A**) before and (**B**) after the addition of large unilamellar vesicles containing cholesterol-PEG_12_-(KIAALKE)_4_. All 26 Texas Red DHPE doped vesicles, marked with white circles, have docked onto the pore-spanning membranes at the end of the time series. Scale bars: 5 μm.

To compare the results of SNARE-mediated fusion with other SNARE mimetics and evaluate the influence of the planar membrane geometry on the overall fusion efficiency, we performed the same experiments with two well-established SNARE fusion mimetics^44^, i.e. the coiled coil forming lipidated peptides cholesterol-PEG_12_-(EIAALEK)_4_ and cholesterol-PEG_12_-(KIAALKE)_4_. Reconstituted into small unilamellar vesicles, the two vesicle populations containing either cholesterol-PEG_12_-(KIAALKE)_4_ or cholesterol-PEG_12_-(EIAALEK)_4_ fuse (Supplementary Fig. 5) as reported previously with very similar synthetic lipidated coiled coil forming peptides^45^,^46^. Next, cholesterol-PEG_12_-(EIAALEK)_4_ was reconstituted into pore-spanning membranes, while cholesterol-PEG_12_-(KIAALKE)_4_ was reconstituted into LUVs using the exact same conditions as reported for the SNAREs. Upon addition of the LUVs to the pore-spanning membranes, vesicles dock onto the membrane (Fig. 7) and remain docked during the entire observation time. No fusion events were observed. Altogether, we analyzed 5 different pore-spanning membrane patches from two different membrane preparations and counted 159 docked vesicles. None of the docked vesicles fused during the observation time. Docking of the vesicles was, however highly specific and did not occur without the reconstitution of the lipopeptides demonstrating that the molecular recognition between the peptide sequences, i.e. the formation of a coiled coil structure takes place.

## Discussion

We successfully established a fusion assay based on solvent-free pore-spanning membranes to monitor protein-mediated fusion events on the single vesicle level. As a proof-of-concept, SNARE-proteins have been chosen to be reconstituted as they are well established to readily fuse membranes^30^. Even though a number of *in vitro* fusion assays have been developed that allow for the detection of single vesicle fusion events^10^,^14^,^15^,^16^, they all rely on solid substrates, which can greatly influence the observed fusion process. If the lipid bilayer is directly attached to the solid substrate, SNARE-mediated fusion events were even observed in the absence of SNAP25^11^,^47^, although numerous *in vivo* and *in vitro* experiments have established that SNAP25 is an essential component of the SNARE-complex in the target membrane to catalyze fusion^2^,^14^,^48^,^49^. Only if the membrane is decoupled from the substrate, SNAP25 dependent fusion has been reported^14^. These results clearly demonstrate that the support of a membrane can greatly influence the fusogenicity and specificity of the entire system. In our setup, we only observed SNAP25 dependent fusion events. No significant interaction of full length synaptobrevin 2 doped LUVs with pore-spanning membranes containing only syntaxin 1A but lacked SNAP25a and the C-terminal soluble fragment of synaptobrevin 2 (residues 49-96) were monitored. In contrast to substrate decoupled solid supported membranes, planar pore-spanning bilayers have the advantage to provide sufficient space for the additionally incoming lipid material, as both leaflets face an aqueous compartment and thus the membrane can be positioned in the third dimension^29^.

Another important aspect that needs to be taken into consideration if a fusion assay is established is membrane curvature. From the comparison of the SNARE-mediated fusion with that of SNARE mimetics, it becomes clear that even though highly curved SUVs doped with the lipopeptides fuse, LUVs do not fuse with a planar pore-spanning membrane indicating that membrane curvature strongly influences the fusion efficiency^50^. This has likewise been observed for other SNARE mimetics^51^,^52^. Curvature might also explain, why the lifetime of the docked vesicles is by a factor of roughly 1000 larger compared to experiments performed on solid supported membranes with SUVs of a mean diameter of 40 nm^14^. In our assay we used LUVs with a mean diameter of 325 nm, which are thermodynamically much more stable^53^, to be able to better resolve each individual vesicle. This notion is corroborated by bulk fusion assays, where the kinetics of fusion is also greatly influenced by the size of the vesicle population, i.e. larger vesicles result in slower fusion kinetics^54^,^55^,^56^. Moreover, if LUVs instead of SUVs are fused with solid supported membranes, the docking time is also significantly increased^50^.

Besides membrane curvature^55^, there are a number of other parameters that are debated in literature to influence the fusion kinetics. The number of SNAREs is controversially discussed and ranges from one to 15 complexes^14^,^57^,^58^,^59^,^60^,^61^. Hernandez *et al.*^50^ recently stated that the actual number of complexes strongly depends on the vesicle size. They found even 23–30 SNARE complexes for >80 nm vesicles, while only three are required in case of small 40 nm sized vesicles.

Another aspect is the lateral membrane tension that has been shown to influence the fusion process including the different fusion states^62^,^63^,^64^,^65^. Even though it is known that the lateral tension of plasma membranes is generally smaller^66^ than those found for pore-spanning membranes^31^,^32^, the local membrane tension at the sites of fusion is not known. It is, however accepted that the cell is capable of regulating vesicle trafficking by plasma membrane tension. While exocytosis is stimulated by high membrane tension, endocytosis stimulated by low membrane tension, increases it^66^. The pore-spanning membrane system allows to modulate the lateral membrane tension by modifying the surface functionalization^32^, which might pave the way for analyzing fusion processes as a function of membrane tension.

In conclusion, the presented membrane fusion system provides several advantages over established fusion assays and is capable of mimicking the natural situation of SNARE-mediated fusion. By time-resolved dual color fluorescence microscopy, it allows resolving individual steps during the fusion process, i.e. docking, intermediate states and full fusion. While the Texas Red DHPE time traces alone provide information similar to lipid dequenching assays^52^, the Oregon Green DHPE time traces further support the different fusion states and can provide additional information about a retarded diffusion of lipids^67^ through the proposed stalk or the formation of flickering fusion pores^9^. Thus, we believe that our system will enable us to quantitatively analyze fusion processes mediated by proteins and mimetics of those and that it will pave the way to bring together *in vitro* and *in vivo* results.

## Methods

### Materials

Porous silicon substrates were received from fluXXion B.V. (Eindhoven, NL). DOPC  (1,2-dioleoyl-*sn*-glycero-3-phosphocholine),  POPE  (1-palmitoyl-2-oleoyl-*sn*-glycero-3-phosphoethanolamine),  and  POPS  (1-palmitoyl-2-oleoyl-*sn*-glycero-3-phospho-L-serine), were purchased from Avanti Polar Lipids (Alabastar, AL, USA). Texas Red DHPE (Texas Red 1,2-dihexadecanoyl-*sn*-glycero-3-phosphoethanolamine, triethylammonium salt), cholsterol as well as 6-mercapto-1-hexanol were obtained from Sigma-Aldrich (Taufkirchen, Germany). Oregon Green 488 DHPE (Oregon Green 488 1,2-dihexadecanoyl-*sn*-glycero-3-phosphoethanolamine) and Atto488 DPPE (Atto488 1,2-dipalmitoyl-*sn*-glycero-3-phosphoethanolamine) were purchased from Molecular Probes (Eugene, OR, USA). KK114 labeled 1,2-dipalmitoyl-*sn*-glycero-3-phosphoethanolamine (DPPE-KK114) was kindly provided by Vladimir Belov (Max-Planck-Institute for Biophysical Chemistry, Göttingen, Germany)^68^. The syntaxin 1A transmembrane domain was synthesized using Fmoc solid phase peptide synthesis^33^ and kindly provided by the Diederichsen group (University of Göttingen, Germany). The lipopeptides cholesterol-PEG_12_-(KIAALKE)_4_ and cholesterol-PEG_12_-(EIAALEK)_4_ were synthesized as described previously^45^.

### Protein expression and purification

All hexahistidine tagged SNARE proteins from *Rattus norvegicus*, i. e. synaptobrevin 2 (residues 1-116, full-length), synaptobrevin 2 (residues 1–96, soluble SNARE binding motif), synaptobrevin 2 (residues 49–96, soluble C-terminal fragment), syntaxin 1A (residues 183–288), SNAP25a (residues 1–206, with all cysteine residues replaced by serine residues) were recombinantly expressed in transformed *Escherichia coli* cells (strain BL21(DE3)) containing the bacterial pET28a expression vector^5^. Purification of the hexahistidine-tagged proteins was carried out as described previously^55^,^69^ using a Ni^2+^-nitrilotriacetic acid affinity chromatography followed by ion exchange chromatography (MonoQ for syntaxin 1A and SNAP25a, MonoS for synaptobrevin and derivatives, Äkta purifying system, GE Healthcare) after cleavage of the hexahistidine tag by thrombin. For the purification of syntaxin 1A and full-length synaptobrevin 2, 1% (*w*/*v*) of the detergent CHAPS was added to the buffer. The ternary SNARE complex composed of syntaxin 1A, synaptobrevin 2 (residues 49-96, soluble C-terminal fragment), and SNAP25a, called ΔN-acceptor complex, was assembled by mixing the three purified proteins with an excess of SNAP25a (1:1:1.5) in presence of 1% CHAPS. After incubation over night at 7 °C, the ΔN-acceptor complex was purified by MonoQ ion exchange chromatography.

### SNARE and SNARE mimetics reconstitution into vesicles

Small unilamellar vesicles containing SNAREs or the lipopeptides were formed upon detergent removal from a micellar protein/lipid/detergent mixture using size exclusion chromatography as described previously^5^. Lipids (DOPC/POPE/POPS/ cholesterol, 5:2:1:2, 0.465 mg total) were mixed from stock solutions in chloroform and dried under a stream of nitrogen. Residual solvent was removed in vacuum (1 hour). The lipid films were solubilized in 50 μL buffer A (20 mM HEPES, 100 mM KCl, 1 mM dithiothreitol (DTT), pH 7.4) containing 100 mM *n*-octyl-β-D-glucopyranoside. After addition of either syntaxin 1A, the ΔN-acceptor complex, or either cholesterol-PEG_12_-(KIAALKE)_4_ or cholesterol-PEG-12-(EIAALEK)_4_ in buffer A with 1% CHAPS to reach a lipid-to-protein ratio of 1,000:1, the mixture was incubated for 0.5 hours on ice. Detergent was removed by size exclusion chromatography on a Sephadex G25 column (Illustra NAP-25, GE Healthcare) equilibrated in buffer A resulting in small unilamellar vesicles^70^.

To form large or giant unilamellar vesicles, multilamellar lipid films were formed from the resulting vesicle suspensions. First, the vesicle suspension was concentrated in a vacuum centrifuge (Eppendorf concentrator 5301) to a volume of about 75 μl and loaded onto a Sephadex G25 column equilibrated in ultrapure water to remove residual detergent and salts. After again concentrating the suspension to 100 μL in a vacuum centrifuge, the suspension was dried overnight at 7 °C in a desiccator over saturated sodium chloride solution (76% relative humidity). For the formation of large unilamellar vesicles (LUVs), the described drying process was performed in a glass test tube, while the electroformation of giant unilamellar vesicles (GUVs) required the drying process to be performed on indium tin oxide (ITO) slides.

To produce LUVs, the proteolipid film was incubated with buffer A for 30 minutes and subsequently vortexed three times every 5 minutes. The suspension was then passed 31 times through a polycarbonate membrane with a nominal pore diameter of 1000 nm using a miniextruder (LiposoFast-Basic, Avestin, Ottawa, CA, USA). Vesicle sizes were determined to be 325 ± 20 nm by dynamic light scattering. For electroformation of GUVs, the proteolipid-covered ITO slides were assembled to form a chamber, which was filled with 200 mM sucrose solution. To grow the GUVs, a sinusoidal voltage (1.6 V_p-p_, 12 Hz) was applied for 3 hours. Functional SNARE and SNARE mimetic reconstitution into LUVs and GUVs was proven by a standard bulk fusion assay (Supplementary Information).

### Preparation of pore-spanning membranes

The top surface of porous silicon nitride substrates with pore diameters of 1.2 μm was first coated with titanium using a sputter coater (15 s, 40 mA, Cressington sputter coater 108auto, Elektronen-Optik-Service, Dortmund, Germany) and subsequently with a gold layer (30 nm) in an evaporation unit under high vacuum with a deposition rate of 0.5–1 nm/s (Bal-Tec Med 020, Balzers, Liechtenstein). The gold surface was functionalized by chemisorption of 6-mercapto-1-hexanol at 8 °C overnight using a 0.1 mM solution in *n-*propanol. Afterwards, the substrates were rinsed with *n*-propanol and buffer A to be mounted in a Teflon chamber filled with 3 mL buffer A. The GUV suspension (25 μL) was added directly onto the porous substrate and incubated for 30 minutes at room temperature. After rinsing, bilayer formation was monitored by fluorescence microscopy.

### Fluorescence correlation spectroscopy (FCS)

For FCS measurements, GUVs composed of DOPC/POPE/POPS/cholesterol, 5:2:1:2 with fluorophore concentrations of 1:10,000 (dye/lipid) for KK114-DPPE and 1:500 (peptide/lipid) for Alexa488-syntaxin 1A were used. These GUVs were spread on functionalized porous silicon substrates to obtain pore-spanning membranes as described. For fluorescence imaging of pore-spanning membranes, a confocal beam-scanning microscopy setup with pulsed two color excitation pulsed-diode lasers (PicoQuant, Berlin, Germany) at 488 nm (pulse length: 80 ps) and 633 nm (pulse length: 80 ps) was used. Emission filters were 540 ± 20 nm for the green channel and 670 ± 30 nm for the red channel. We used a 100× oil immersion objective (NA = 1.42, Leica, Wetzlar, Germany), and avalanche single photon counting detectors (APD, SPCM-AQR-13-FC, Perkin Elmer Optoelectronics).

To study the dynamics of syntaxin 1A and the lipids in the pore-spanning membranes, we applied FCS. From the time resolved fluorescence intensity traces *F(t)* the autocorrelation function *G*(*τ*) is calculated (eq. (3)):
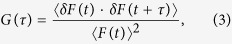
where *F* is the fluorescence intensity, *t* the time, δ*F*(*t*) the temporal fluorescence intensity fluctuations, and *τ* the lag time; the angular brackets refer to time averaging. Since we monitor lateral diffusion in the plane of the membrane, a two-dimensional diffusion model was fitted to the autocorrelation function (eq. (4)):
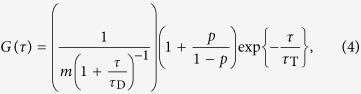
where *m* is the average number of fluorescently labeled molecules in the detected volume and *p* is the fraction of labeled molecules, which convert to the dark triplet state. *τ*_D_ is the average transit time of molecules moving through the observation volume and *τ*_T_ is the average time the labeled molecule remains in the triplet state. From the diffusion time *τ*_D_, we obtain the diffusion coefficient *D* (eq. (5)):
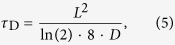
where *L is* the lateral width of the focal detection volume at half maximum fluorescence intensity. For Alexa488-syntaxin 1A *L* was 200 nm, and for KK114-DPPE *L* was 250 nm. For each diffusion constant, at least 20 pore-spanning membranes from three independent reconstitutions were analyzed.

### Confocal laser scanning fluorescence microscopy imaging

An upright confocal laser scanning microscope (Zeiss LSM 710, Zeiss, Jena, Germany) equipped with a water immersion objective with 63× magnification (NA = 1.0, Zeiss, W Plan-Apochromat) was used for fluorescence imaging. A 488 nm argon-laser was used to excite both, the red and the green fluorescent dye simultaneously. Emission light was spectrally separated and collected by photo multiplier tubes for Oregon Green and Atto488 in the range of 500–560 nm and for Texas Red at 600–680 nm.

### Single vesicle fusion detection and data analysis

For monitoring fusion events of single vesicles with pore-spanning membranes, images of 256 × 256 pixels^2^ related to an area of 40 × 40 μm^2^ were recorded with a time resolution of about 120 ms/frame over a period of 300 s (2500 images) after the addition of 0.5 μl vesicle suspension to the pore-spanning membrane. To automatically detect the single vesicle fusion events, a global threshold was subtracted from the red channel intensity to locate the vesicles diffusing in proximity to the pore-spanning membrane with a minimum size of two pixels. The individually numbered particles were followed by a particle-tracking algorithm. At the position, where the vesicle remains fixed on the membrane surface, i.e. the vesicle has been docked to the membrane, a region of interest (ROI) with a radius of 3 pixels was defined around the center of mass of the particle and the intensity of the red and the green channel in the ROI was integrated and read out as a function of time. The generated time-resolved fluorescence intensity traces were used to analyze the individual fusion events. Resulting histograms for docking and hemifusion times were statistically corrected to account for the finite time window of data acquisition according to eq. (6):
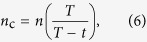
with *n* the originally determined events, *n*_c_ the corrected ones and *T,* the time window of data acquisition (308 s).

## Additional Information

**How to cite this article**: Schwenen, L. L. G. *et al.* Resolving single membrane fusion events on planar pore-spanning membranes. *Sci. Rep.* **5**, 12006; doi: 10.1038/srep12006 (2015).

## Supplementary Material

Supplementary Information

Supplementary Movie 1

Supplementary Movie 2

## Figures and Tables

**Figure 1:**
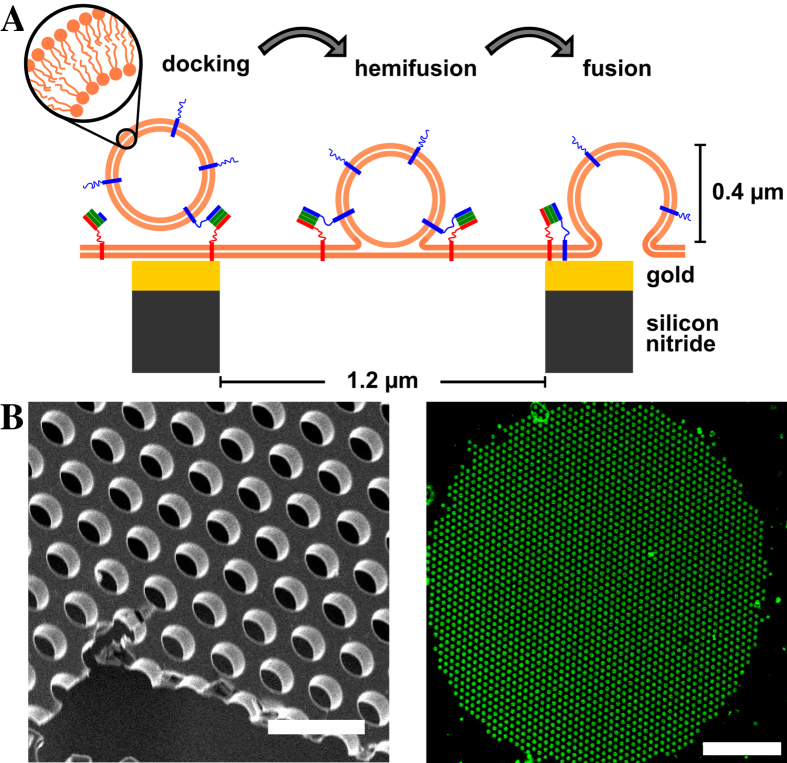
(**A**) Schematic drawing of the model system for SNARE mediated membrane fusion based on pore-spanning membranes. SNARE proteins are shown in red (syntaxin 1A), green (SNAP25) and blue (synaptobrevin 2). Membranes are depicted as thick orange lines. (**B**) Scanning electron micrograph of a porous silicon nitride substrate. Scale bar: 3 μm. (**C**) Pore-spanning membrane patch obtained from spreading a giant unilamellar vesicle composed of DOPC/POPE/POPS/cholesterol (5:2:1:2) and doped with 1 mol % Oregon Green DHPE on a gold/6-mercapto-1-hexanol-functionalized substrate. Scale bar: 20 μm.

## References

[b1] Weber T. *et al.* SNAREpins: Minimal machinery for membrane fusion. Cell 92, 759–772 (1998). 9529252 10.1016/s0092-8674(00)81404-x

[b2] Schuette C. G. *et al.* Determinants of liposome fusion mediated by synaptic SNARE proteins. Proc. Natl. Acad. Sci. U.S.A. 101, 2858–2863 (2004). 14981239 10.1073/pnas.0400044101 PMC365710

[b3] Ji H. *et al.* Protein determinants of SNARE-mediated lipid mixing. Biophys. J. 99, 553–560 (2010). 20643074 10.1016/j.bpj.2010.04.060 PMC2905075

[b4] Li F. *et al.* A half-zippered SNARE complex represents a functional intermediate in membrane fusion. J. Am. Chem. Soc. 136, 3456–3464 (2014). 24533674 10.1021/ja410690m PMC3985920

[b5] Pobbati A. V. & Stein A., Fasshauer D. N- to C-terminal SNARE complex assembly promotes rapid membrane fusion. Science 313, 673–676 (2006). 16888141 10.1126/science.1129486

[b6] Malsam J. *et al.* Complexin arrests a pool of docked vesicles for fast Ca^2+^-dependent release. EMBO J. 31, 3270–3281 (2012). 22705946 10.1038/emboj.2012.164 PMC3411073

[b7] Tareste D., Shen J., Melia T. J. & Rothman J. E. SNAREpin/Munc18 promotes adhesion and fusion of large vesicles to giant membranes. Proc. Natl. Acad. Sci. U.S.A. 105, 2380–2385 (2008). 18268324 10.1073/pnas.0712125105 PMC2268145

[b8] Anzai K., Masumi M., Kawasaki K. & Kirino Y. Frequent fusion of liposomes to a positively charged planar bilayer without calcium ions. J. Biochem. 114, 487–491 (1993). 7506250 10.1093/oxfordjournals.jbchem.a124204

[b9] Chanturiya A., Chernomordik L. V. & Zimmerberg J. Flickering fusion pores comparable with initial exocytotic pores occur in protein-free phospholipid bilayers. Proc. Natl. Acad. Sci. U.S.A. 94, 14423–14428 (1997). 9405628 10.1073/pnas.94.26.14423 PMC25008

[b10] Fix M. *et al.* Imaging single membrane fusion events mediated by SNARE proteins. Proc. Natl. Acad. Sci. U.S.A. 101, 7311–7316 (2004). 15123811 10.1073/pnas.0401779101 PMC409915

[b11] Liu T., Tucker W. C., Bhalla A., Chapman E. R. & Weisshaar J. C. SNARE-driven, 25-millisecond vesicle fusion *in vitro*. Biophys. J. 89, 2458–2472 (2005). 16055544 10.1529/biophysj.105.062539 PMC1366745

[b12] Wang T., Smith E. A., Chapman E. R. & Weisshaar J. C. Lipid mixing and content release in single-vesicle, SNARE-driven fusion assay with 1-5 ms resolution. Biophys. J. 96, 4122–4131 (2009). 19450483 10.1016/j.bpj.2009.02.050 PMC2712201

[b13] Wessels L., Elting M. W., Scimeca D. & Weninger K. Rapid membrane fusion of individual virus particles with supported lipid bilayers. Biophys. J. 93, 526–538 (2007). 17449662 10.1529/biophysj.106.097485 PMC1896232

[b14] Domanska M. K., Kiessling V., Stein A., Fasshauer D. & Tamm L. K. Single vesicle millisecond fusion kinetics reveals number of SNARE complexes optimal for fast SNARE-mediated membrane fusion. J. Biol. Chem. 284, 32158–32166 (2009). 19759010 10.1074/jbc.M109.047381 PMC2797286

[b15] Domanska M. K., Kiessling V. & Tamm L. K. Docking and fast fusion of synaptobrevin vesicles depends on the lipid compositions of the vesicle and the acceptor SNARE complex-containing target membrane. Biophys. J. 99, 2936–2946 (2010). 21044591 10.1016/j.bpj.2010.09.011 PMC2965956

[b16] Floyd D. L., Ragains J. R., Skehel J. J., Harrison S. C. & Van Oijen A. M. Single-particle kinetics of influenza virus membrane fusion. Proc. Natl. Acad. Sci. U.S.A. 105, 15382–15387 (2008). 18829437 10.1073/pnas.0807771105 PMC2556630

[b17] Yoon T. Y., Okumus B., Zhang F., Shin Y. K. & Ha T. Multiple intermediates in SNARE-induced membrane fusion. Proc. Natl. Acad. Sci. U.S.A. 103, 19731–19736 (2006). 17167056 10.1073/pnas.0606032103 PMC1698870

[b18] Diao J. *et al.* A single-vesicle content mixing assay for SNARE-mediated membrane fusion. Nat. Commun. 1, 54 (2010). 20975723 10.1038/ncomms1054 PMC3518844

[b19] Diao J. J. *et al.* A single vesicle-vesicle fusion assay for *in vitro* studies of SNAREs and accessory proteins. Nat. Protoc. 7, 921–934 (2012). 22582418 10.1038/nprot.2012.020 PMC4410872

[b20] Otterstrom J. & Van Oijen A. M. Visualization of membrane fusion, one particle at a time. Biochemistry 52, 1654–1668 (2013). 23421412 10.1021/bi301573w

[b21] Wagner M. L. & Tamm L. K. Tethered polymer-supported planar lipid bilayers for reconstitution of integral membrane proteins: silane-polyethyleneglycol-lipid as a cushion and covalent linker. Biophys. J. 79, 1400–1414 (2000). 10969002 10.1016/S0006-3495(00)76392-2 PMC1301034

[b22] Mey I., Steinem C. & Janshoff A. Biomimetic functionalization of porous substrates: towards model systems for cellular membranes. J. Mater. Chem. 22, 19348–19356 (2012).

[b23] Reimhult E. & Kumar K. Membrane biosensor platforms using nano- and microporous supports. Trends Biotechnol. 26, 82–89 (2008). 18191259 10.1016/j.tibtech.2007.11.004

[b24] Warkiani M. E. *et al.* Isoporous micro/nanoengineered membranes. ACS Nano 7, 1882–1904 (2013). 23442009 10.1021/nn305616k

[b25] Zagnoni M. Miniaturised technologies for the development of artificial lipid bilayer systems. Lab Chip 12, 1026–1039 (2012). 22301684 10.1039/c2lc20991h

[b26] Römer W. *et al.* Channel activity of a viral transmembrane peptide in micro-BLMs: Vpu1-32 from HIV-1. J. Am. Chem. Soc. 126, 16267–16274 (2004). 15584764 10.1021/ja0451970

[b27] Weiskopf D., Schmitt E. K., Klühr M. H., Dertinger S. K. & Steinem C. Micro-BLMs on highly ordered porous silicon substrates: rupture process and lateral mobility. Langmuir 23, 9134–9139 (2007). 17655338 10.1021/la701080u

[b28] Kaufeld T., Steinem C. & Schmidt C. F. Microporous device for local electric recordings on model lipid bilayers. J. Phys. D: Appl. Phys. 48, 025401 (2015).

[b29] Höfer I. & Steinem C. A membrane fusion assay based on pore-spanning lipid bilayers. Soft Matter 7, 1644–1647 (2011).

[b30] Jahn R. & Fasshauer D. Molecular machines governing exocytosis of synaptic vesicles. Nature 490, 201–207 (2012). 23060190 10.1038/nature11320 PMC4461657

[b31] Kocun M., Lazzara T. D., Steinem C. & Janshoff A. Preparation of solvent-free, pore-spanning lipid bilayers: modeling the low tension of plasma membranes. Langmuir 27, 7672–7680 (2011). 21619014 10.1021/la2003172

[b32] Kuhlmann J. W., Mey I. P. & Steinem C. Modulating the lateral tension of solvent-free pore-spanning membranes. Langmuir 30, 8186–8192 (2014). 24950370 10.1021/la5019086

[b33] Van Den Bogaart G. *et al.* Membrane protein sequestering by ionic protein-lipid interactions. Nature 479, 552–555 (2011). 22020284 10.1038/nature10545 PMC3409895

[b34] Sieber J. J., Willig K. I., Heintzmann R., Hell S. W. & Lang T. The SNARE motif is essential for the formation of syntaxin clusters in the plasma membrane. Biophys. J. 90, 2843–2851 (2006). 16443657 10.1529/biophysj.105.079574 PMC1414554

[b35] Sieber J. J. *et al.* Anatomy and dynamics of a supramolecular membrane protein cluster. Science 317, 1072–1076 (2007). 17717182 10.1126/science.1141727

[b36] Lazzara T. D., Carnarius C., Kocun M., Janshoff A. & Steinem C. Separating attoliter-sized compartments using fluid pore-spanning lipid bilayers. ACS Nano 5, 6935–6944 (2011). 21797231 10.1021/nn201266e

[b37] Kiessling V. & Tamm L. K. Measuring distances in supported bilayers by fluorescence interference-contrast microscopy: polymer supports and SNARE proteins. Biophys. J. 84, 408–418 (2003). 12524294 10.1016/S0006-3495(03)74861-9 PMC1302622

[b38] Bacia K., Schuette C. G., Kahya N., Jahn R. & Schwille P. SNAREs prefer liquid-disordered over “raft” (liquid-ordered) domains when reconstituted into giant unilamellar vesicles. J. Biol. Chem. 279, 37951–37955 (2004). 15226320 10.1074/jbc.M407020200

[b39] Przybylo M. *et al.* Lipid diffusion in giant unilamellar vesicles is more than 2 times faster than in supported phospholipid bilayers under identical conditions. Langmuir 22, 9096–9099 (2006). 17042516 10.1021/la061934p

[b40] Weiss K. *et al.* Quantifying the diffusion of membrane proteins and peptides in black lipid membranes with 2-focus fluorescence correlation spectroscopy. Biophys. J. 105, 455–462 (2013). 23870266 10.1016/j.bpj.2013.06.004 PMC3714877

[b41] Chi Y. S. *et al.* Polymeric rulers: Distance-dependent emission behaviors of fluorophores on flat gold surfaces and bioassay platforms using plasmonic fluorescence enhancement. Adv. Funct. Mater. 18, 3395–3402 (2008).

[b42] Aravanis A. M., Pyle J. L., Harata N. C. & Tsien R. W. Imaging single synaptic vesicles undergoing repeated fusion events: kissing, running, and kissing again. Neuropharmacology 45, 797–813 (2003). 14529718 10.1016/s0028-3908(03)00310-1

[b43] Harata N. C., Aravanis A. M. & Tsien R. W. Kiss-and-run and full-collapse fusion as modes of exo-endocytosis in neurosecretion. J. Neurochem. 97, 1546–1570 (2006). 16805768 10.1111/j.1471-4159.2006.03987.x

[b44] Robson Marsden H., Elbers N. A., Bomans P. H. H., Sommerdijk N. A. J. M. & Kros A. A reduced SNARE model for membrane fusion. Angew. Chem. Int. Ed. 48, 2330–2333 (2009). 10.1002/anie.200804493 19222065

[b45] Versluis F. *et al.* *In situ* modification of plain liposomes with lipidated coiled coil forming peptides induces membrane fusion. J. Am. Chem. Soc. 135, 8057–8062 (2013). 23659206 10.1021/ja4031227

[b46] Zheng T. *et al.* Controlling the rate of coiled coil driven membrane fusion. Chem. Comm. 49, 3649–3651 (2013). 23531995 10.1039/c3cc38926j

[b47] Bowen M. E., Weninger K., Brunger A. T. & Chu S. Single molecule observation of liposome-bilayer fusion thermally induced by soluble N-ethyl maleimide sensitive-factor attachment protein receptors (SNAREs). Biophys. J. 87, 3569–3584 (2004). 15347585 10.1529/biophysj.104.048637 PMC1304822

[b48] Blasi J. *et al.* Botulinum neurotoxin A selectively cleaves the synaptic protein SNAP-25. Nature 365, 160–163 (1993). 8103915 10.1038/365160a0

[b49] Washbourne P. *et al.* Genetic ablation of the t-SNARE SNAP-25 distinguishes mechanisms of neuroexocytosis. Nat. Neurosci. 5, 19–26 (2002). 11753414 10.1038/nn783

[b50] Hernandez J. M., Kreutzberger A. J. B., Kiessling V., Tamm L. K. & Jahn R. Variable cooperativity in SNARE-mediated membrane fusion. Proc. Natl. Acad. Sci. U.S.A. 111, 12037–12042 (2014). 25092301 10.1073/pnas.1407435111 PMC4143004

[b51] Chan Y. H., Van Lengerich B., Boxer S. G. Effects of linker sequences on vesicle fusion mediated by lipid-anchored DNA oligonucleotides. Proc. Natl. Acad. Sci. U.S.A. 106, 979–984 (2009). 19164559 10.1073/pnas.0812356106 PMC2633564

[b52] Van Lengerich B., Rawle R. J., Bendix P. M. & Boxer S. G. Individual vesicle fusion events mediated by lipid-anchored DNA. Biophys. J. 105, 409–419 (2013). 23870262 10.1016/j.bpj.2013.05.056 PMC3714935

[b53] Lichtenberg D. *et al.* Effect of surface curvature on stability, thermodynamic behavior, and osmotic activity of dipalmitoylphosphatidylcholine single lamellar vesicles. Biochemistry 20, 3462–3467 (1981). 6894860 10.1021/bi00515a024

[b54] Cypionka A. *et al.* Discrimination between docking and fusion of liposomes reconstituted with neuronal SNARE-proteins using FCS. Proc. Natl. Acad. Sci. U.S.A. 106, 18575–18580 (2009). 19843696 10.1073/pnas.0906677106 PMC2764736

[b55] Hernandez J. M. *et al.* Membrane fusion intermediates via directional and full assembly of the SNARE complex. Science 336, 1581–1584 (2012). 22653732 10.1126/science.1221976 PMC3677693

[b56] McMahon H. T., Kozlov M. M. & Martens S. Membrane curvature in synaptic vesicle fusion and beyond. Cell 140, 601–605 (2010). 20211126 10.1016/j.cell.2010.02.017

[b57] Shi L. *et al.* SNARE proteins: one to fuse and three to keep the nascent fusion pore open. Science 335, 1355–1359 (2012). 22422984 10.1126/science.1214984 PMC3736847

[b58] Karatekin E. *et al.* A fast, single-vesicle fusion assay mimics physiological SNARE requirements. Proc. Natl. Acad. Sci. U.S.A. 107, 3517–3521 (2010). 20133592 10.1073/pnas.0914723107 PMC2840481

[b59] Montecucco C., Schiavo G., Pantano S. SNARE complexes and neuroexocytosis: how many, how close? Trends Biochem. Sci. 30, 367–372 (2005). 15935678 10.1016/j.tibs.2005.05.002

[b60] Sinha R., Ahmed S., Jahn R. & Klingauf J. Two synaptobrevin molecules are sufficient for vesicle fusion in central nervous system synapses. Proc. Natl. Acad. Sci. U.S.A. 108, 14318–14323 (2011). 21844343 10.1073/pnas.1101818108 PMC3161593

[b61] Van Den Bogaart G. *et al.* One SNARE complex is sufficient for membrane fusion. Nat. Struct. Mol. Biol. 17, 358–364 (2010). 20139985 10.1038/nsmb.1748 PMC2924150

[b62] Shillcock J. C. & Lipowsky R. Tension-induced fusion of bilayer membranes and vesicles. Nat. Mater. 4, 225–228 (2005). 15711550 10.1038/nmat1333

[b63] Markosyan R. M., Melikyan G. B. & Cohen F. S. Tension of membranes expressing the hemagglutinin of influenza virus inhibits fusion. Biophys. J. 77, 943–952 (1999). 10423439 10.1016/S0006-3495(99)76945-6 PMC1300385

[b64] Bretou M. *et al.* Cdc42 controls the dilation of the exocytotic fusion pore by regulating membrane tension. Mol. Biol. Cell 25, 3195–3209 (2014). 25143404 10.1091/mbc.E14-07-1229 PMC4196869

[b65] Chernomordik L. V. & Kozlov M. M. Mechanics of membrane fusion. Nat. Struct. Mol. Biol. 15, 675–683 (2008). 18596814 10.1038/nsmb.1455 PMC2548310

[b66] Gauthier N. C., Masters T. A. & Sheetz M. P. Mechanical feedback between membrane tension and dynamics. Trends Cell Biol. 22, 527–535 (2012). 22921414 10.1016/j.tcb.2012.07.005

[b67] Bao C., Pähler G., Geil B. & Janshoff A. Optical fusion assay based on membrane-coated spheres in a 2D assembly. J. Am. Chem. Soc. 135, 12176–12179 (2013). 23915348 10.1021/ja404071z

[b68] Wurm C. A. *et al.* Novel red fluorophores with superior performance in STED microscopy. Optical Nanoscopy 1, 1–7 (2012).

[b69] Fasshauer D., Antonin W., Margittai M., Pabst S. & Jahn R. Mixed and non-cognate SNARE complexes. Characterization of assembly and biophysical properties. J. Biol. Chem. 274, 15440–15446 (1999). 10336434 10.1074/jbc.274.22.15440

[b70] Seddon A. M., Curnow P. & Booth P. J. Membrane proteins, lipids and detergents: not just a soap opera. Biochim. Biophys. Acta 1666, 105–117 (2004). 15519311 10.1016/j.bbamem.2004.04.011

