# Supplementary material for: Resolving single membrane fusion events on planar pore-spanning membranes
Source: Sci Rep. 2015 Jul 13;5:12006. doi: 10.1038/srep12006 (PMC4499801; doi:10.1038/srep12006)
Supplement: Supplementary Information [file srep12006-s1.pdf]

## Supplementary Information

# Resolving single membrane fusion events on planar pore-spanning membranes

Lando L. G. Schwenen,[1] Raphael Hubrich,[1] Dragomir Milovanovic,[2] Burkhard Geil,[3] Jian Yang,[4] Alexander Kros,[4] Reinhard Jahn,[2] Claudia Steinem[1]*

[1] Institute for Organic and Biomolecular Chemistry, University of Göttingen, Tammannstr. 2, 37077 Göttingen, Germany,

[2] Max-Planck-Institute for Biophysical Chemistry, Am Fassberg 11, 37077 Göttingen, Germany,

[3] Institute for Physical Chemistry, University of Göttingen, Tammannstr. 6, 37077 Göttingen, Germany,

[4] Leiden Institute of Chemistry - Supramolecular and Biomaterials Chemistry, Leiden University, Einsteinweg 55, 2333 CC Leiden, The Netherlands.

**FCS measurements of Alexa488-syntaxin 1A.** To investigate the diffusion behavior of the incorporated syntaxin 1A in the pore-spanning membrane, fluorescence correlation spectroscopy (FCS) was performed using Alexa488-syntaxin 1A (Fig. 1).

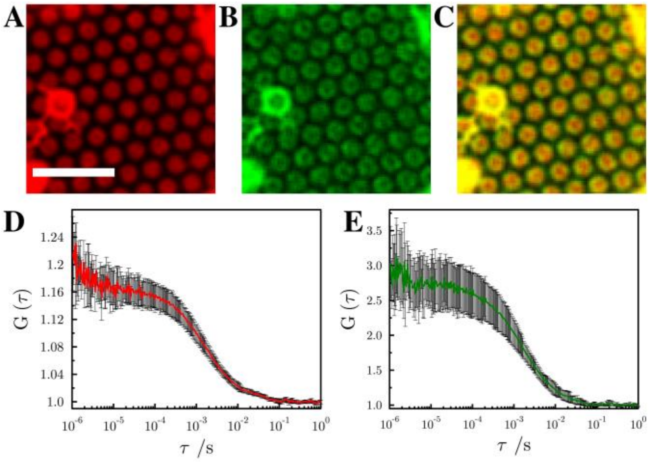


**Fig. 1.** Fluorescence micrographs of pore-spanning membranes composed of DOPC/POPE/POPS/cholesterol (5:2:1:2) on a 6-mercapto-1-hexanol functionalized gold surface. The images show fluorescence signals of (**A**) DPPE-KK114, (0.01 mol%), (**B**) Alexa488-syntaxin 1A (0.2 mol%) and (**C**) an overlay of (A) and (B). Scale bar: 5 µm. Autocorrelation curves of the performed FCS measurements with excitation wavelengths of 633 nm (DPPE-KK114, D) and 488 nm (Alexa488-syntaxin 1A, E). Fitting eq. (4) to the autocorrelation curves provide diffusion coefficients of 7.4 ± 0.3 µm²/s (SD) for DPPE-KK114 and 2.3 ± 0.5 µm²/s (SD) for Alexa488-syntaxin 1A. For each molecule the averaged curves of 5 measurements from two independent reconstitutions are shown.

**FLIP experiments.** As the mobility of the membrane components localized on the pore rims could not be analyzed by FCS measurements due to the quenching of the fluorescence intensity by the underlying gold surface, fluorescence loss in photobleaching (FLIP) experiments were performed using a confocal laser scanning microscope (Zeiss LSM 710, Zeiss, Jena, Germany). GUVs for pore-spanning membrane preparations were produced as described in the Methods section, however with an increased amount of Atto647N-syntaxin 1-transmembrane domain (1:6,000, peptide/lipid). A small region of a membrane patch (ROI **1**) was bleached simultaneously with exciting the fluorescence at wavelengths of 488 (Atto488 DPPE, green) and 650 nm (Atto647N-syntaxin 1-TMD, red) for 300-800 s. After the bleaching time, the fluorescence of the membrane patch was monitored for another 100 s.

Fig 2. shows the result of a FLIP experiment for Atto488 DPPE. The bleaching spot after the bleaching time (Fig. 2B, ROI **1**) shows a constant normalized fluorescence intensity of 0.53, indicating successful bleaching of the fluorophores in the spot, while the normalized fluorescence intensity of the entire membrane patch (Fig. 2B, ROI **2**) is 0.56, being only about 6 % larger than the fluorescence intensity in the bleaching spot. This demonstrates that the Atto488 DPPE lipids can diffuse into and out of the bleaching spot and are thus in continuous exchange with the entire membrane patch.

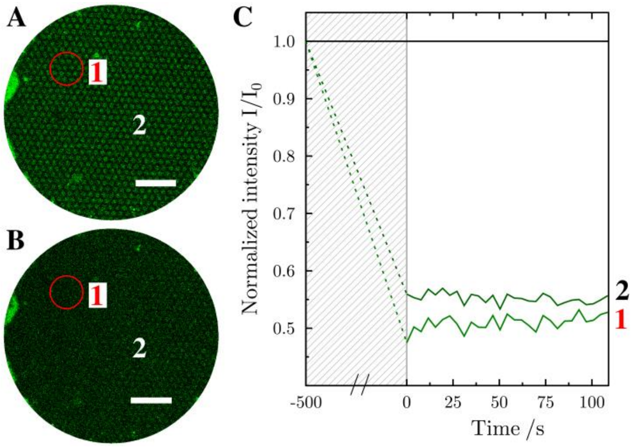


**Fig. 2.** Fluorescence micrographs of an Atto488 DPPE-doped membrane patch (**A**) before and (**B**) after ($t_{bleach}$ = 500 s) bleaching a small region (ROI **1**) within the entire membrane patch (ROI **2**). (**C**) Intensity courses in the two ROIs as indicated. Fluorescence intensities are normalized to a references ROI ($I_0(t)$) on a different membrane patch on the full image. The shaded area corresponds to the bleaching time.

Fig. 3 shows the result of a FLIP experiment for Atto647N-syntaxin 1-TMD from the same membrane patch as shown in Fig. 2. The bleaching spot after the bleaching time (Fig. 3B, ROI **1**) shows a decrease in normalized fluorescence intensity to 0.78 indicating bleaching. The bleaching is less effective than for Atto488 DPPE because of the higher photostability of the fluorescent dye and the lower power output of the bleaching laser (488 nm Ar ion laser 25 mW, 633 nm HeNe laser 5 mW). After the bleaching time, the normalized fluorescence intensity slightly recovers to 0.89 as a result of some non-bleached Atto647N-syntaxin 1-helices diffusing back into the bleaching spot. The normalized fluorescence intensity of the entire membrane patch (Fig. 3B, ROI **2**) is also decreased ($I/I_0$ = 0.93) as a result of Atto647N-syntaxin 1-helices being in continuous exchange with the entire membrane patch.

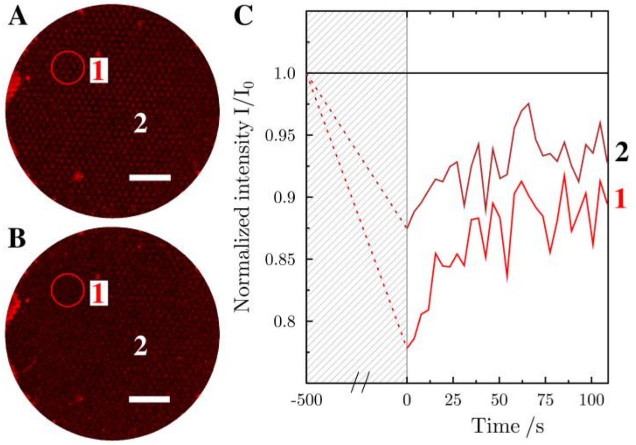


**Fig. 3.** Fluorescence micrographs of an Atto647N-syntaxin 1-TMD-doped membrane patch (**A**) before and (**B**) after ($t_{\text{bleach}}$ = 500 s) bleaching a small region (ROI **1**) within the entire membrane patch (ROI **2**). (**C**) Intensity courses in the two ROIs as indicated. Fluorescence intensities are normalized to a references ROI ($I_0(t)$) on a different membrane patch on the full image. The shaded area corresponds to the bleaching time.

**Vesicle bulk fusion assays with SNAREs.** To analyze SNARE protein functionality at each step of the reconstitution process, bulk vesicle fusion experiments were performed with vesicles prepared as described in the Methods section[1]. A fluorometer (Jasco FP-6500 Spectrofluorometer) set to an excitation wavelength of 501 nm and an emission wavelength of 607 nm with a 3 nm band width was used. To 600 µL of buffer A (20 mM HEPES, 100 mM KCl, 1 mM dithiothreitol (DTT), pH 7.4) heated to 37 °C, 100 µL of Oregon Green DHPE doped vesicles (1 mol%) containing the ΔN-acceptor complex were added under constant stirring. After a stable baseline was reached, Texas Red DHPE (1 mol%) doped vesicles containing synaptobrevin 2 (100 µL) were added. Fig. 4A shows the intensity course of the Texas Red emission after vesicle addition. The increase in intensity reflects mixing of the lipids of the two vesicle populations as the two fluorescent dyes come in close proximity resulting in efficient Förster resonance energy transfer (FRET). As a control experiment, the ΔN-acceptor complex containing vesicles were incubated with the soluble SNARE binding motif of synaptobrevin 2 (residues 1-96) prior to the experiment. The same lipid mixing assay was performed with large unilamellar vesicles (LUVs doped with 1 mol% Texas Red DHPE and synaptobrevin 2) and giant unilamellar vesicles (GUVs doped with 1 mol% Oregon Green DHPE and the ΔN-acceptor complex) (Fig. 4B). A slower kinetics is observed as reported previously[2].

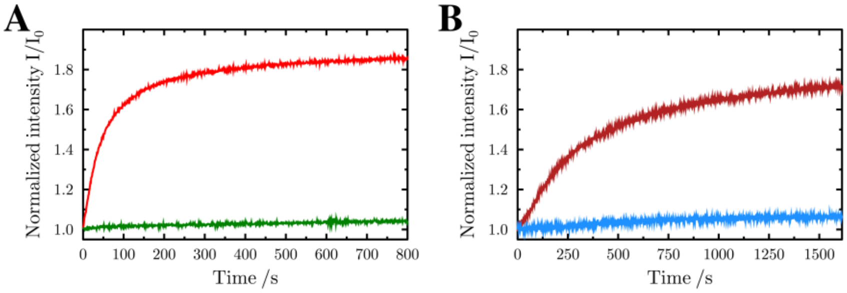


**Fig. 4.** Fluorescence intensity time courses. (**A**) Lipid mixing of small unilamellar vesicles (SUVs) with reconstituted SNARE proteins (red) and control experiment with blocked ΔN-acceptor complex (green). (**B**) Lipid mixing of GUVs containing the ΔN-acceptor complex and LUVs containing synaptobrevin 2 (dark red) and control experiment with blocked ΔN-acceptor complex (blue). The fluorescence intensity is normalized to $I_0 = I(t = 0)$.

**Vesicle bulk fusion assays with SNARE mimetics.** To analyze the functionality of the lipopeptides cholesterol-PEG$_{12}$-(KIAALKE)$_4$ and cholesterol-PEG$_{12}$-(EIAALEK)$_4$ at each step of the reconstitution process, the same bulk fusion experiments as described above were performed with vesicles prepared as described in the Methods section. The fluorometer was set to an excitation wavelength of 490 nm and an emission wavelength of 607 nm with a 3 nm band width. To 650 µL of buffer A heated to 37 °C, 150 µL of Atto488 DPPE (1 mol%) doped small unilamellar vesicles (SUVs) containing cholesterol-PEG$_{12}$-(EIAALEK)$_4$ were added under constant stirring. After a stable baseline was reached, Texas Red DHPE (1 mol%) doped SUVs containing cholesterol-PEG$_{12}$-(KIAALKE)$_4$ (150 µL) were added. Fig. 5A shows the intensity course of the Texas Red emission after vesicle addition demonstrating lipid mixing of the SUVs as expected from previous studies[3, 4]. Fig. 5B shows the same lipid mixing assay performed with large unilamellar vesicles (LUVs doped with 1 mol% Texas Red DHPE and cholesterol-PEG$_{12}$-(KIAALKE)$_4$) and giant unilamellar vesicles (GUVs doped with 1 mol% Atto488 DPPE and cholesterol-PEG$_{12}$-(EIAALEK)$_4$). In contrast to the SNARE-mediated fusion, the intensity increase is strongly reduced.

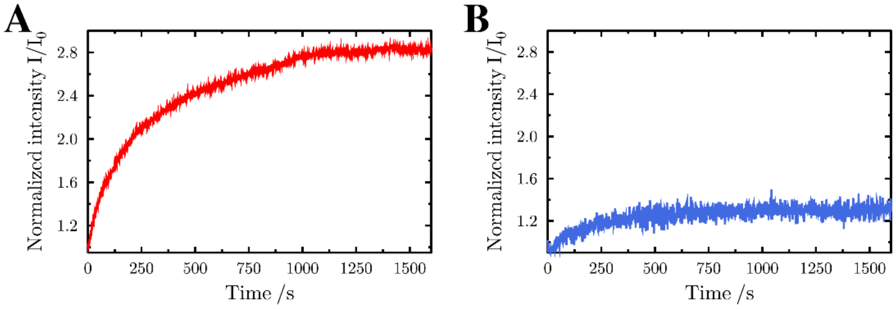


**Fig. 5.** Fluorescence intensity time courses. (**A**) Lipid mixing of small unilamellar vesicles (SUVs) with reconstituted cholesterol-PEG$_{12}$-(EIAALEK)$_4$ and cholesterol-PEG$_{12}$-(KIAALKE)$_4$. (**B**) Lipid mixing of GUVs containing cholesterol-PEG$_{12}$-(EIAALEK)$_4$ and LUVs containing cholesterol-PEG$_{12}$-(KIAALKE)$_4$ (blue). The fluorescence intensity is normalized to $I_0 = I(t = 0)$.

**Movies of vesicle fusion on pore-spanning membranes.** Movie 1 (1000 images) shows a characteristic time series collected as described in the Methods section. LUVs (Texas Red DHPE doped, red) diffuse atop the pore-spanning membranes (Oregon Green DHPE doped, green).

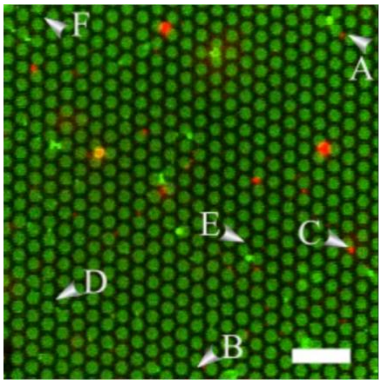


**Movie 1.** Still image of Movie 1 showing 1000 frames of one time series in two channels. Green: Oregon Green DHPE doped pore-spanning membranes with reconstituted ΔN-acceptor complex; red: Texas Red DHPE doped LUVs with reconstituted synaptobrevin 2. The movie represents a timespan of 120 s in real time. Intensity courses of the vesicles marked with A-F are depicted in Fig. 6. Scale bar: 5 µm.

During the course of the movie some vesicles dock and subsequently hemifuse or fuse with the pore-spanning membrane. Intensity courses of some randomly picked vesicles (positions A-F) are depicted in Fig. 6 and marked in the movie.

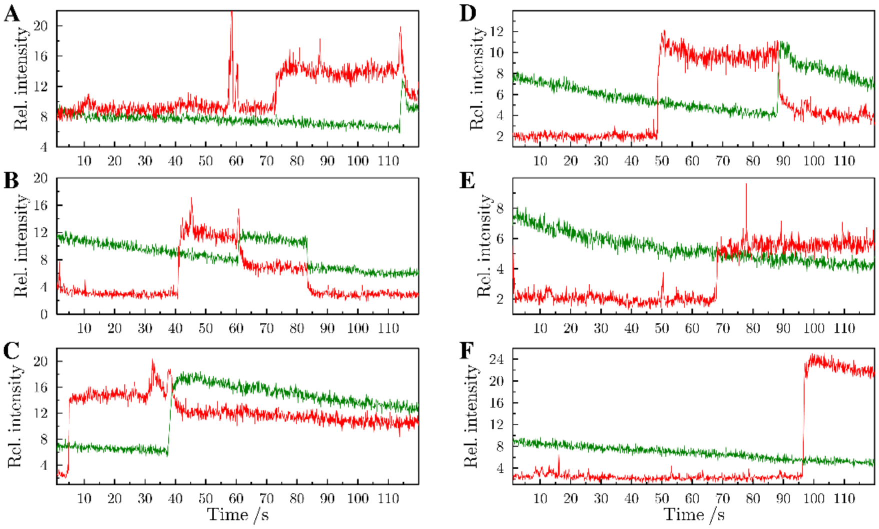


**Fig. 6.** Intensity courses of the events marked in Movie 1. Based on the criteria discussed in the main text and detailed below the events are classified as docked only (**E**,**F**), hemifused (**A**,**C** and **D**), and fully fused (**B**).

Movie 2 shows a time series recorded under the same conditions as Movie 1 with the only difference that prior to GUV spreading they were incubated with the soluble SNARE binding motif of synaptobrevin 2 (residues 1-96) as described in the Methods section. After addition of LUVs, they diffuse atop the pore-spanning membranes but do not dock. The same result was obtained, when the pore-spanning membranes lacked SNAP25a.

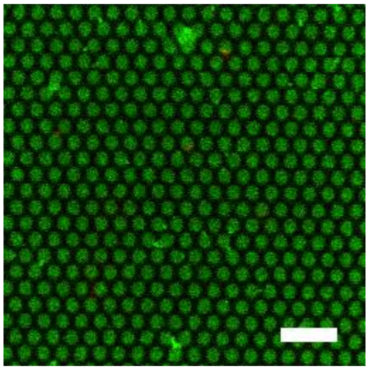

**Movie 2.** Still image of Movie 2 showing 1000 frames of one time series in two channels. Green: Oregon Green DHPE doped pore-spanning membranes with reconstituted ΔN-acceptor complex blocked with the soluble SNARE binding motif of synaptobrevin 2 (residues 1-96); red: Texas Red DHPE doped LUVs with reconstituted synaptobrevin 2. The LUVs diffuse atop the pores-spanning membranes but do not dock. The movie represents a timespan of 109 s in real time. Scale bar: 5 µm.

**Classification of fusion events.** To classify the events into docking, hemifusion and full fusion, we used the following criteria as visualized by the idealized fluorescence intensity time traces of the OG and TR fluorescence (Fig 7.).

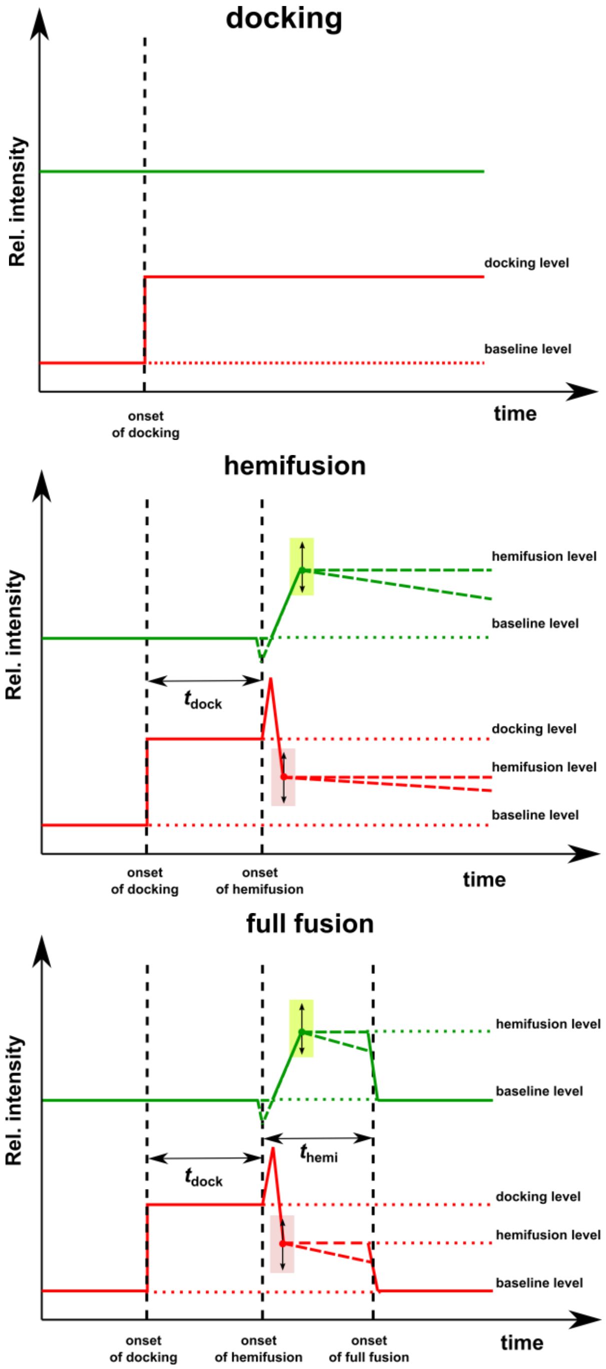


**Fig. 7.** The solid lines represent the time traces of the TR (red) and OG (green) fluorescence intensities. Dotted lines serve as a guide to the eye to visualize the corresponding levels. Broken lines demonstrate that there is a variation in the time traces. The broken vertical lines show the time points to determine $t_{dock}$ and $t_{hemi}$. $t_{dock}$ is defined as the time period between the onset of docking of the vesicle and the onset of hemifusion, i.e. the time period, where the vesicle remains in the docked state. $t_{hemi}$ is the time period, where the vesicle remains in an intermediate, hemifused state. The shaded boxes indicate that the hemifusion levels vary as a result of the different vesicle sizes located in a defined ROI and the position of vesicle fusion on the pore-spanning membrane (pore rime of freestanding part).

# References


1. Pobbati AV, Stein A, Fasshauer D. N- to C-terminal SNARE complex assembly promotes rapid membrane fusion. *Science* **313**, 673-676 (2006).
2. Hernandez JM*, et al.* Membrane fusion intermediates via directional and full assembly of the SNARE complex. *Science* **336**, 1581-1584 (2012).
3. Versluis F*, et al.* In situ modification of plain liposomes with lipidated coiled coil forming peptides induces membrane fusion. *J. Am. Chem. Soc.* **135**, 8057-8062 (2013).
4. Zheng T*, et al.* Controlling the rate of coiled coil driven membrane fusion. *Chem. Comm.* **49**, 3649-3651 (2013).
